# Supplementary material for: Optimised Electroporation for Loading of Extracellular Vesicles with Doxorubicin
Source: Pharmaceutics. 2021 Dec 24;14(1):38. doi: 10.3390/pharmaceutics14010038 (PMC8780628; doi:10.3390/pharmaceutics14010038)
Supplement: Supplementary file 1 [file pharmaceutics-14-00038-s001.zip › pharmaceutics-1455291-supplementary.pdf]

pharmaceutics

Supplementary Materials: Optimised Electroporation for Loading of Extracellular Vesicles with Doxorubicin

Angus J. Lennaárd, Doste R. Mamand, Rim Jawad Wiklander, Samir EL Andaloussi and Oscar P.B. Wiklander

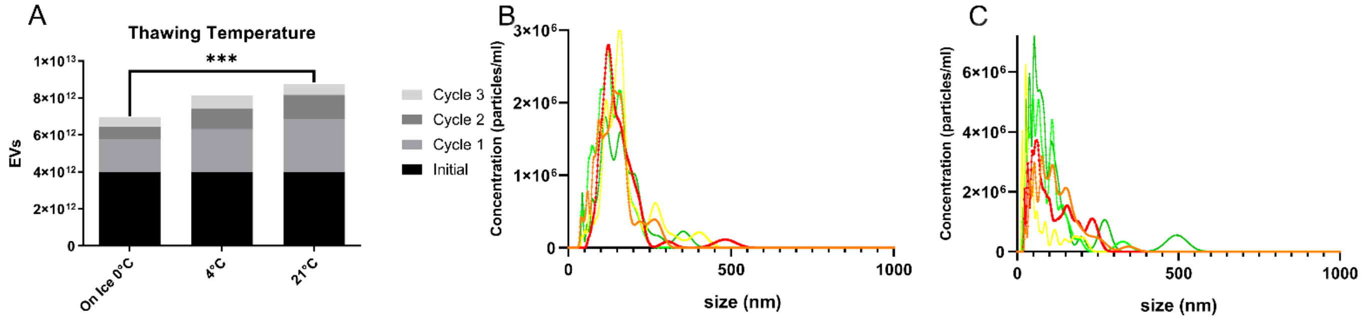

Figure S1. Thawing of EVs at different temperatures. (**A**) The total EV concentration in all samples after three cycles of freezing and thawing in three different temperatures including RT around 21 °C, 4 °C and on ice. (**B**) Size distribution curve of samples thawed at RT after 3 cycles. (**C**) Size distribution curve of samples thawed on ice after 3 cycles. Significance calculated by ANOVA and is illustrated as follows: ***P <.001.

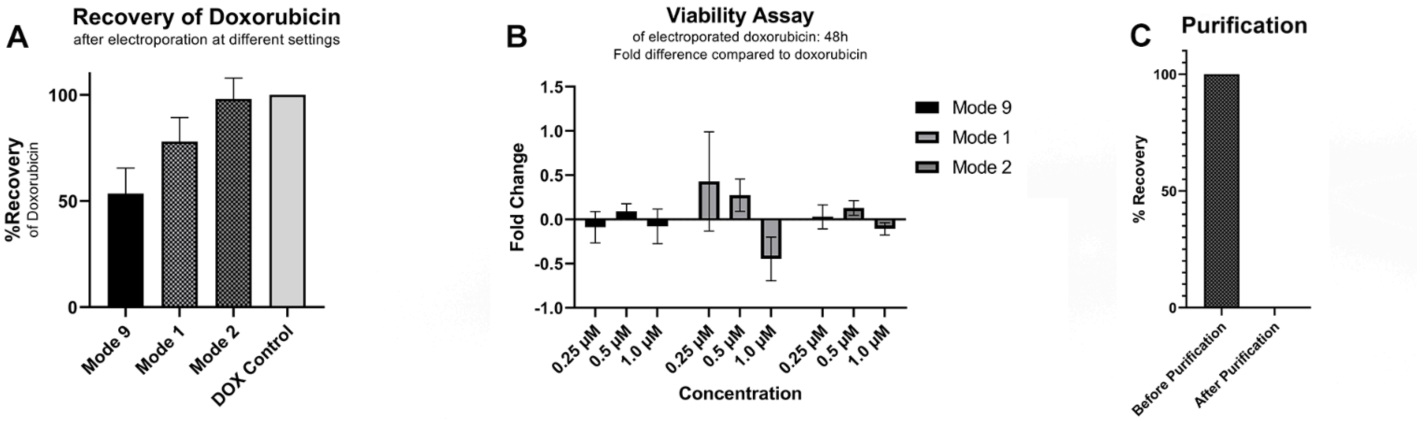

Figure S2. Removal and effect of unbound doxorubicin (**A**) The recovery of naked doxorubicin was determined by fluorometer post-electroporation in different conditions. (**B**) In vitro viability assay demonstrating the altered potency of naked doxorubicin post-electroporation in different conditions. Represented in fold change to untreated naked doxorubicin. (**C**) The effective removal of naked doxorubicin after loading according to the optimized protocol in absence of EVs, by size exclusion chromatography and subsequently ultrafiltration.

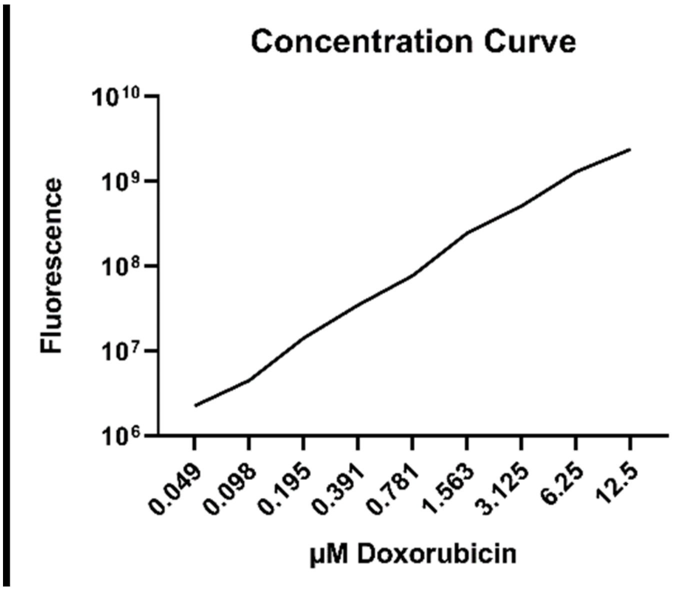

Figure S3. Quantification of doxorubicin. A concentration curve illustrating the relationship between the fluorescent readout and the concentration of doxorubicin, demonstrating a linear relationship within the visualised range.
